# Epidemiologie, Diagnostik und Therapie der Aufmerksamkeitsdefizit-Hyperaktivitäts-Störung (ADHS) im höheren Lebensalter

**DOI:** 10.1007/s00115-023-01548-7

**Published:** 2023-09-25

**Authors:** Peter Praus, Alexander Moldavski, Barbara Alm, Oliver Hennig, Michael Rösler, Wolfgang Retz

**Affiliations:** 1 Zentralinstitut für Seelische Gesundheit, Klinik für Psychiatrie und Psychotherapie, J5, 68159 Mannheim, Deutschland; 2 Pfalzklinikum für Neurologie und Psychiatrie, Klinik für Forensische Psychiatrie, Weinstraße 100, 76889 Klingenmünster, Deutschland; 3 Institut für Gerichtliche Psychologie und Psychiatrie der Universität des Saarlandes, Universitätsklinikum des Saarlandes, 66421 Homburg/Saar, Deutschland; 4 Klinik für Psychiatrie und Psychotherapie, Universitätsmedizin Mainz, Untere Zahlbacher Straße 8, 55131 Mainz, Deutschland

**Keywords:** Hyperkinetische Störung, Senioren, Versorgung, Screening, Demenz, Hyperkinetic disorder, Elderly, Care, Screening, Dementia

## Abstract

Aktuelle Studien belegen eine vergleichsweise hohe Prävalenz der Aufmerksamkeitsdefizit-Hyperaktivitäts-Störung (ADHS) bis ins höhere Lebensalter. Ältere Betroffene leiden unter einer hohen Belastung mit psychiatrischer und somatischer Komorbidität sowie erheblichen Einschränkungen ihres psychosozialen Funktionsniveaus und subjektiven Wohlbefindens. Die differenzialdiagnostische Abgrenzung gegenüber neurodegenerativen Erkrankungen ist besonders in dieser Altersgruppe schwierig. Die vorliegende narrative Übersichtsarbeit will den aktuellen Wissensstand zur Epidemiologie der ADHS im höheren Lebensalter und zu möglichen Zusammenhängen zwischen ADHS und dem Risiko für Neurodegeneration zusammenfassen. Darüber hinaus werden Empfehlungen zur Diagnostik der ADHS im höheren Lebensalter und Behandlungsoptionen dargestellt.

## Abstract (en)

Current studies demonstrate a comparably high prevalence of attention deficit-hyperactivity disorder (ADHD) in advanced age. Older people affected by ADHD suffer from a severe burden of psychiatric and somatic comorbidities as well as substantial impairment of social functioning and subjective well-being. The diagnostic differentiation from neurodegenerative diseases is particularly difficult in this age group. This narrative review summarizes the current knowledge about the epidemiology of ADHD in advanced age and possible relationships between ADHD and the risk for neurodegeneration. Furthermore, recommendations for diagnostics and treatment options of ADHD in advanced age are presented.

## Einleitung

Die Aufmerksamkeitsdefizit-Hyperaktivitäts-Störung (ADHS) ist eine neuropsychiatrische Störung, die sich in der Kindheit und Jugend manifestiert und in vielen Fällen bis ins Erwachsenenalter persistiert [38]. Nicht selten hat die Störung über die Lebensspanne, sofern keine adäquate Behandlung erfolgt, verheerende Auswirkungen auf die psychosoziale Anpassung, das Wohlbefinden und die mentale Gesundheit der Betroffenen. Genetische Faktoren – vermutlich ergänzt durch Umwelteinflüsse – spielen für das Risiko für das Auftreten einer ADHS eine bedeutende Rolle. Eine aktuelle Metaanalyse weist eine gepoolte Prävalenz der ADHS mit Persistenz im Erwachsenenalter von 4,61 % aus [37], mit erheblichen Qualitätsunterschieden zwischen einzelnen Studien und niedrigeren Prävalenzen in Ländern mit hohem Einkommen (3,25 %) und höheren Prävalenzen in Ländern mit mittlerem und niedrigem Einkommen (8,0 %). In älteren Studien wird die Prävalenz der ADHS im Erwachsenenalter mit Werten zwischen 2,5 % [34] und 2,8 % [12] bis 4,4 % [17] angegeben. Somit stellt sich die Frage, wie im höheren Lebensalter eine klinisch relevante ADHS nachgewiesen werden kann und welche spezifischen Charakteristika und Bedürfnisse die Betroffenen aufweisen. In entwickelten Ländern der westlichen Welt tragen neben körperlichen Erkrankungen die Alzheimer-Demenz und depressive Störungen in der Altersgruppe ab 70 Jahren signifikant zu Funktionseinbußen und Krankheitslast bei [4]. Global betrachtet sind in dieser Hinsicht auch Angststörungen in der Altersgruppe ab 50 Jahren relevant [5]. Passend hierzu zeigen aktuelle Daten aus der Bundesrepublik Deutschland, dass neurologische Erkrankungen, also v. a. Schlaganfälle und Demenzen, in den Alterskohorten ab 65 Jahren signifikant zur Zahl der durch Tod verlorenen Lebensjahre beitragen, während beispielsweise der Anteil von durch Sterblichkeit verlorenen Lebensjahren („years of life lost“ [YLL]), der durch Substanzkonsumstörungen verursacht wird, im höheren Lebensalter zurückgeht [42]. Hieraus ergibt sich, dass die Diagnostik und Differenzialdiagnostik der ADHS im höheren Lebensalter primär neurodegenerative Erkrankungen, aber auch affektive Störungen und Angsterkrankungen berücksichtigen muss. Die folgende Arbeit soll einen orientierenden Überblick zur Epidemiologie der ADHS im höheren Lebensalter bieten. Zudem sollen Empfehlungen zur Diagnostik und Therapie der ADHS in dieser Altersgruppe unter Berücksichtigung relevanter Komorbiditäten und Differenzialdiagnosen diskutiert werden.

## Methoden

**Figure 1:**
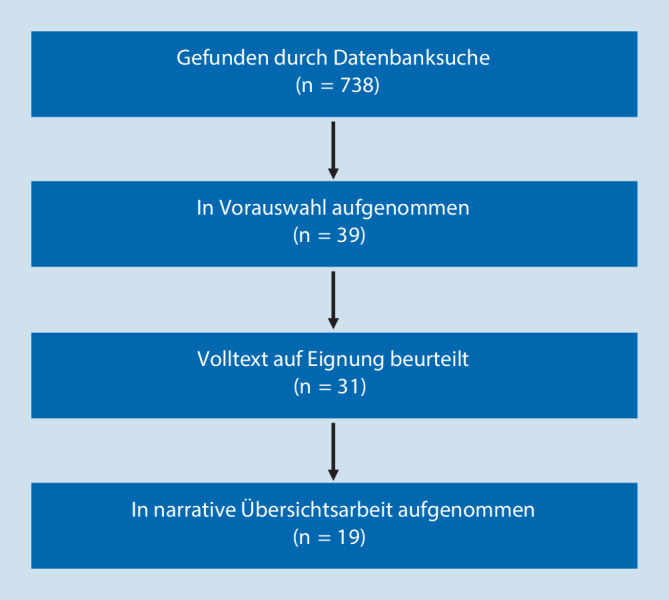

Für die Literaturrecherche wurden die Datenbanken Medline (via PubMed), PsycINFO/PsycARTICLES und Google Scholar genutzt (letzter Abruf am 15.07.2023). Neben dem Stichwort „ADHS“ bzw. „ADHD“ wurden folgende Suchbegriffe verwendet: Alter, Senium, elderly, advanced age, old age, older adults, dementia, neurodegeneration, treatment, stimulants. Die so gefundenen Veröffentlichungen wurden anhand der Überschriften und Abstracts hinsichtlich ihrer Relevanz gesichtet und es wurden die Publikationen ausgewählt, die im Hinblick auf das gewählte Thema bedeutsam erschienen. Ausgeschlossen wurden in einem weiteren Schritt Studien, die keine Probanden ab einem Alter von 60 Jahren eingeschlossen hatten. Ebenso wurden Fallberichte, Fallserien und die Artikel ausgeschlossen, die sich nicht auf Epidemiologie, Diagnostik und Behandlung der ADHS im höheren Lebensalter bezogen. Abschließend wurden die Referenzlisten der ausgewählten Publikationen zusätzlich nach relevanten Veröffentlichungen durchsucht. Einen Überblick über das Vorgehen bietet Abb. 1.

## Epidemiologie der ADHS im höheren Lebensalter

Bislang vorliegende Untersuchungen gehen überwiegend von einer Prävalenz der ADHS von ca. 2–3 % im höheren Lebensalter aus [8]. Bei der Mehrzahl dieser Studien ist jedoch eine ausgeprägte methodische Heterogenität zu berücksichtigen, mit generell höheren Punktprävalenzen, wenn die Symptomatik mit standardisierten Skalen und Fragebögen erfasst wurde, und niedrigeren, wenn klinische ADHS-Diagnosen und Behandlungsdaten herangezogen wurden. Darüber hinaus zeichnen sich niedrigere Prävalenzen in der Altersgruppe ab 60 Jahren ab (0,77 %), wenn zusätzlich eine Persistenz der Symptomatik seit der Kindheit und Jugend berücksichtigt wird [37]. Eine Metaanalyse von Song und Kollegen kam darüber hinaus zu dem Schluss, dass eine generelle Abnahme der Prävalenz der ADHS über die Lebensspanne zu beobachten ist [37]. Eine aktuelle Studie aus Deutschland, die auch retrospektiv eine ADHS-Symptomatik in der Kindheit erfasste, fand eine geschätzte Prävalenz der ADHS von 3,1 % in der Altersgruppe von 40 bis 59 und 2,1 % in der Altersgruppe von 60 bis 80 Jahren [27].

Zusammenfassend ist die Datenlage zur Prävalenz der ADHS im höheren Lebensalter aufgrund methodischer Beschränkungen noch als vergleichsweise unzureichend zu bezeichnen. Die überwiegende Anzahl der Studien legt jedoch nahe, dass eine ADHS bis ins höhere Lebensalter in relevantem Umfang anhand charakteristischer Symptome und Funktionsstörungen diagnostiziert werden kann. Auch die ADHS im höheren Lebensalter ist mit typischen Defiziten der Aufmerksamkeitsfunktionen und des Arbeitsgedächtnisses assoziiert, die partiell durch eine höhere Belastung mit depressiven Symptomen erklärt werden können [32]. Darüber hinaus beklagen ältere Patienten mit ADHS häufiger interpersonelle Konflikte und eine ausgeprägte biografische Akkumulation negativer Lebensereignisse [31], leiden in Abhängigkeit von der Schwere der Symptomatik häufiger unter depressiven und Angstsymptomen [25] sowie chronischen körperlichen Erkrankungen [33], beschreiben eine nachhaltige Beeinträchtigung der Lebensqualität und des sozialen Funktionsniveaus durch die Symptomatik [28] und leiden häufiger unter Einsamkeit [23] als nicht von ADHS betroffene Individuen ihrer Altersgruppe.

## ADHS und Neurodegeneration

### ADHS und Demenzrisiko

Aus longitudinalen US-amerikanischen Krankenhausdaten geht hervor, dass ein schwer ausgeprägter („severe“) ADHS-Phänotyp innerhalb eines Zeitraums von zehn Jahren das Risiko, mit der Diagnose einer Alzheimer- (AD) oder Lewy-body-Demenz (LBD) aus einer Krankenhausbehandlung entlassen zu werden, deutlich erhöhte [13]. Wurden die Daten für den Faktor Diabetes korrigiert, blieb lediglich eine Assoziation zwischen ADHS und dem Auftreten einer LBD bestehen, woraus die Autoren schlussfolgerten, dass ein möglicherweise erhöhtes AD-Risiko bei ADHS-Patienten im Wesentlichen über metabolische Faktoren vermittelt wird. Dies bestätigt Ergebnisse einer Fall-Kontroll-Studie von Golimstok und Kollegen, die 2011 erstmals bei 109 Patienten mit LBD eine erhöhte Prävalenz (47,8 %) von ADHS-Symptomen mit Persistenz im Erwachsenenalter feststellte, nicht aber bei Patienten mit AD [16]. Eine retrospektive Kohortenstudie aus Taiwan konnte ein 3,4-fach erhöhtes Risiko von Erwachsenen mit ADHS nachweisen, an einer Demenz zu erkranken [41]. Bemerkenswert ist, dass dieses Risiko bei ADHS-Patienten bereits vor einer Altersgrenze von 55 Jahren erhöht war und für sämtliche Demenzformen außer AD nachgewiesen werden konnte. Demgegenüber legen die Ergebnisse einer aktuellen Registerstudie aus Schweden Hinweise auf eine transgenerationale familiäre Häufung von Demenzerkrankungen, insbesondere auch einer AD mit frühem Beginn, bei Probanden mit einer ADHS nahe [43]. Dies bestätigt die Ergebnisse einer weiteren schwedischen Registerstudie, die Daten von mehr als 3,5 Mio. Individuen systematisch auswertete. Bei Individuen mit einer ADHS-Diagnose wurde hier ein um den Faktor drei erhöhtes Risiko, an einer leichten kognitiven Störung („mild cognitive impairment“ [MCI]) bzw. einer Demenz, einschließlich einer AD, zu erkranken, nachgewiesen [9]. Dieses Risiko wurde signifikant durch zusätzlich diagnostizierte psychiatrische Störungen, z. B. Suchterkrankungen und affektive Störungen, jedoch nur in geringem Umfang durch internistische Begleiterkrankungen, z. B. Bluthochdruck, Diabetes mellitus und Übergewicht, moduliert. Eine aktuelle Registerstudie aus Schweden [36] konnte darüber hinaus ein erhöhtes Risiko für eine leichte kognitive Störung oder eine Demenzerkrankung im Alter bei in der Vergangenheit verurteilten Straftätern, also einer Personengruppe mit vergleichsweise hoher ADHS-Prävalenz [30], in Abhängigkeit von der Länge der Haftstrafe und dem Schweregrad der begangenen Straftaten nachweisen. ADHS-Diagnosen wurden dabei jedoch nicht erfasst. Direkte Zusammenhänge zwischen einer ADHS-Diagnose, Delinquenz und Demenzrisiko konnten somit bisher nicht nachgewiesen werden.

Zusammenfassend weist der aktuelle Forschungsstand darauf hin, dass eine ADHS das Risiko, im Alter an einem MCI, einer Altersdemenz und ggf. auch einer präsenilen Demenz zu erkranken, signifikant erhöht. Psychiatrische Begleiterkrankungen tragen neben metabolischen Risikofaktoren in bedeutsamem Umfang zu diesem Risiko bei.

### ADHS und andere neurodegenerative Erkrankungen

Ferner konnte kürzlich ein um den Faktor 4 erhöhtes Risiko von ADHS-Patienten, die im Erwachsenenalter mit Stimulanzien behandelt worden waren, an einem Parkinson-Syndrom (PS) zu erkranken, belegt werden [6]. In derselben Studie zeigte sich auch ein erhöhtes Risiko für andere Erkrankungen der Basalganglien und des Kleinhirns bei erwachsenen Patienten mit ADHS. Untersuchungen zu einer möglichen gemeinsamen Grundlage von ADHS und PS konnten jedoch bislang keinen genetischen Zusammenhang nachweisen [15].

Zusammenfassend erscheint es somit naheliegender, zusätzliche Risikofaktoren, die häufig mit einer ADHS assoziiert sind und sich nachteilig auf ein gesundes Altern des Gehirns auswirken können, in Überlegungen zur Beziehung zwischen einer ADHS mit Persistenz über die Lebensspanne und dem Risiko, im Alter an neurodegenerativen Prozessen zu erkranken, einzubeziehen. Dies sind im Einzelnen: gesundheitsschädliches Verhalten, z. B. Störungen durch Substanzkonsum [11], niedriger sozioökonomischer Status und psychiatrische Komorbidität [35], psychosoziale Faktoren wie soziale Isolation [1] etc. Obgleich anzunehmen ist, dass kognitiven Störungen im Alter und ADHS trotz mancher symptomatischer Überlappungen, beispielsweise bei Defiziten der exekutiven Funktionen, in den meisten Fällen unterschiedliche pathophysiologische Prozesse zugrunde liegen, so erwachsen hieraus dennoch besondere Anforderungen an die differenzialdiagnostische Abgrenzung der ADHS von möglichen Prodromalsyndromen einer Demenz.

## Diagnostik

Auch die Diagnostik der ADHS im höheren Lebensalter sollte grundsätzlich den aktuellen S3-Leitlinien zur Diagnostik der ADHS im Erwachsenenalter folgen (AWMF-Registernummer 028-045). Sofern standardisierte Rating-Skalen und Interviews zum Einsatz kommen, liegt derzeit für deutschsprachige Patienten ab einem Alter von 60 Jahren lediglich eine Validierungsstudie für die Homburger ADHS-Skalen für Erwachsene (HASE) vor [29], die eine zeitökonomische und valide Erfassung der Symptomatik mit zufriedenstellender Sensitivität und Spezifität im höheren Lebensalter erlauben. Auch die deutsche Kurzfassung der Conners’ Adult ADHD Rating Scales (CAARS) [3] hat sich im Kontext von Studien zur Diagnostik einer ADHS im höheren Lebensalter bewährt [27]. Eine besondere Herausforderung dürfte darin bestehen, die Symptomatik und ihre Auswirkungen über die Lebensspanne bei älteren Patienten außerhalb von Selbstberichten, die anfällig für einen sog. „recall bias“ sein können, zu rekonstruieren. Daher sollten, wo immer möglich, fremde Informationsquellen, z. B. ärztliche Befundberichte, Schulzeugnisse und Angaben von Angehörigen, in die Anamneseerhebung einbezogen werden. Diese können auch zusätzliche klinische Hinweise auf eine differenzialdiagnostisch zu berücksichtigende, erst im Alter erworbene kognitive Störung geben. Darüber hinaus ist einer Häufung körperlicher Erkrankungen mit zunehmendem Lebensalter umfassend Rechnung zu tragen. Hier erscheint die Durchführung einer internistisch-neurologischen Untersuchung, eines Elektrokardiogramms und einer Laboruntersuchung des Blutes mit Differenzialblutbild, Transaminasen, Nierenfunktionsparametern etc. sinnvoll. Im Zweifel sollten auch weiterführende Untersuchungen bei Ärzten anderer Fachdisziplinen großzügig angeboten werden, z. B. eine kranielle Kernspintomographie, eine Langzeitblutdruckmessung bzw. eine fachkardiologische Mitbeurteilung.

Eine klare diagnostische Unterscheidung zwischen einer ADHS und einem MCI anhand neuropsychologischer Testprofile ist derzeit nicht möglich [22]. Möglicherweise deuten aber ein „Speicherdefizit“ und Beeinträchtigungen des „semantic retrieval“ eher auf das Vorliegen eines MCI hin [2]. Demgegenüber könnten Einschränkungen des Arbeitsgedächtnisses in Korrelation mit einem charakteristischen klinischen Befund eher auf eine ADHS hinweisen [32]. Es ist allerdings anzumerken, dass diese Defizite in Funktionen des Arbeitsgedächtnisses bei älteren ADHS-Patienten partiell durch eine Belastung mit depressiven Symptomen erklärt werden konnten. Ferner ist zu bedenken, dass bis zu 20 % älterer Patienten mit ADHS keine klar umrissenen neuropsychologischen Defizite aufweisen [39]. Gerade ältere Patienten mit einer ADHS zeigten sowohl im Quer- als auch im Längsschnitt eine erhöhte Belastung mit depressiven Symptomen und Angstsymptomen [25]. Daher sollte auch bei Patienten mit einer „Altersdepression“ bei der Anamneseerhebung überprüft werden, ob der Symptomatik eine bislang nicht diagnostizierte ADHS zugrunde liegen könnte. V. a. Angaben zum Verlauf der Beschwerden (chronisch vs. episodisch), weiteren typischen Begleiterkrankungen, die mit einer ADHS assoziiert sind (z. B. Substanzgebrauchsstörungen), und charakteristischen Persönlichkeitsmerkmalen (z. B. niedriger Selbstwert) können im Rahmen der Differenzialdiagnostik hilfreich sein [18, 24]. Dass eine ADHS erstmals im höheren Lebensalter diagnostiziert wird, kann einerseits durch ein Versagen von Kompensationsreserven, z. B. im Zusammenhang mit zunehmenden körperlichen Einschränkungen, erklärt werden. Andererseits können Änderungen der Lebensbedingungen, z. B. der Umzug in eine Heimeinrichtung, zu Beschränkungen von Freiheiten und Handlungsspielräumen führen und Anpassungen an eine teilweise fremdbestimmte Lebensführung erforderlich machen und somit zu einer Demaskierung der ADHS-Symptomatik beitragen. Demgegenüber ist im höheren Alter und mit dem Ausscheiden aus dem Erwerbsleben in der Regel von einem Nachlassen der im Alltag zu bewältigenden Stressoren auszugehen, sodass sich nachlassendes Kompensationsvermögen nicht zwingend nachteilig auswirken muss.

## Behandlung

### Therapie mit Stimulanzien

Laut einer aktuellen Untersuchung wird weit weniger als die Hälfte aller ADHS-Patienten im höheren Lebensalter behandelt [8], was die Frage aufwirft, ob dies Ausdruck einer Unterversorgung ist oder einer spezifischen Anpassung der Behandlungsstrategie an die Erfordernisse während des letzten Lebensabschnitts nach dem Ende des Erwerbslebens entspricht. Naturalistische Untersuchungen legen nahe, dass auch im höheren Lebensalter eine Behandlung mit Stimulanzien mit beherrschbaren Risiken durchgeführt werden kann, von der Patienten im Hinblick auf Alltagsbewältigung und allgemeines Wohlbefinden auch profitieren [40]. Hierbei ist grundsätzlich auf körperliche Begleiterkrankungen, z. B. des Herz-Kreislauf-Systems, aber auch psychiatrische Komorbiditäten zu achten, die den Einsatz von Stimulanzien bei dieser Patientengruppe beschränken können. Die bislang größte retrospektive Beobachtungsstudie zur Behandlung älterer ADHS-Patienten mit Stimulanzien an insgesamt 113 Probanden mit einem Altersrange von 55 bis 79 Jahren konnte jedoch bei 65 % der Patienten ein überwiegend positives Ansprechen auf eine Stimulanzientherapie feststellen [26]. Zur Behandlung wurden überwiegend retardiertes Methylphenidat und Dexmethylphenidat eingesetzt. In einigen Fällen kamen aber auch unretardiertes Methylphenidat und Dexamfetamin zum Einsatz. Diese Patienten berichteten u. a. von einer Verbesserung der Konzentration, einer Stabilisierung der Stimmung, verbessertem Schlaf, reduzierter Impulsivität und verbesserter Funktionalität im Alltag. Eine Behandlung mit Methylphenidat war auf Gruppenebene mit einem geringen Gewichtsverlust und einem leichten Anstieg der Herzfrequenz assoziiert. Bei Patienten mit einem kardiovaskulären Risikoprofil konnte nach der Einleitung einer wirksamen Behandlung mit Methylphenidat weder eine statistisch signifikante Änderung des systolischen und diastolischen Blutdrucks noch der Herzfrequenz beobachtet werden. Neben kardiovaskulären Nebenwirkungen führten andererseits Schlafstörungen, Angst- und depressive Symptome zu einem vorzeitigen Abbruch der Therapie.

Zusammenfassend scheint angesichts dieser Studienergebnisse unter engmaschigen Kontrollen klinischer Parameter wie Gewicht, Herzfrequenz, Blutdruck und EKG sowie der Beachtung internistischer Erkrankungen auch im höheren Lebensalter eine wirksame medikamentöse Therapie der ADHS mit vertretbarem Risikoprofil durchführbar zu sein. Ergänzend sollten dann aus der Sicht der Autoren auch regelmäßig Bestimmungen des Augeninnendrucks zur Glaukomprophylaxe durchgeführt werden. Bei Männern sollten anamnestische Hinweise auf eine Prostatahyperplasie mit Restharnbildung Anlass zu einer urologischen Mitbeurteilung vor der Einleitung einer Stimulanzientherapie geben. Es muss jedoch betont werden, dass nur wenige Daten zu den pharmakologischen Eigenschaften von Stimulanzien im höheren Lebensalter bei Patienten mit körperlichen Begleiterkrankungen vorliegen. Mit zunehmendem Alter und der Anzahl der Begleiterkrankungen steigt auch das Risiko für eine Polypharmazie, womit sich die Wahrscheinlichkeit für das Auftreten von Arzneimittelinteraktionen deutlich erhöht. Ferner können physiologische Alterungsprozesse zu Änderungen von Pharmakokinetik und -dynamik führen, auf die bei einer medikamentösen Behandlung der ADHS im höheren Lebensalter Rücksicht genommen werden muss. In der Bundesrepublik Deutschland sind Stimulanzien bis zum 60. Lebensjahr zur Behandlung der adulten ADHS zugelassen; eine Verschreibung soll gemäß Herstellerangaben bei älteren Menschen nicht erfolgen. Derzeit ist also eine Stimulanzientherapie älterer Patienten mit ADHS lediglich auf der Grundlage einer Off-label-Verordnung möglich, obwohl die oben skizzierte Datenlage durchaus auch in dieser Altersgruppe eine insgesamt gute Verträglichkeit und Wirksamkeit vermuten lässt. Eine am 06.02.2018 veröffentlichte Stellungnahme der damaligen Leitungsgruppe des zentralen adhs-netzes (www.zentrales-adhs-netz.de) plädierte trotz des erweiterungsbedürftigen empirischen Wissens dafür, dass eine Behandlung mit Methylphenidat oder Atomoxetin auch bei Senioren indiziert sein kann.

Zusammenfassend sollte die Indikationsstellung für eine Behandlung mit Stimulanzien im höheren Lebensalter somit unter engmaschiger fachärztlicher Beobachtung psychiatrischer und kardiovaskulärer Nebenwirkungen sowie in enger Abstimmung mit Ärzten anderer Fachgebiete, v. a. der Inneren Medizin und Allgemeinmedizin, nach ausführlicher Aufklärung des Patienten über Chancen und Risiken einer derartigen Therapie in seiner Altersgruppe erfolgen. Einen Überblick über relevante Kontraindikationen für eine Therapie mit Stimulanzien gibt Tab. 1. Unter Berücksichtigung dieser Einschränkungen ist auch im höheren Lebensalter eine medikamentöse Behandlung der ADHS mit vertretbarem Risiko durchführbar.

**Table 1:** 

Bekannte Überempfindlichkeit
Glaukom
Phäochromozytom
Prostatahyperplasie mit Restharnbildung
Behandlung mit einem irreversiblen MAO-Hemmer
Schlecht kontrollierte bipolar-affektive Störung, schwere Depression, Manie, Schizophrenie
Herz-Kreislauf-Erkrankungen (maligne Hypertonie, Herzinsuffizienz, pAVK, hämodynamisch wirksame Herzfehler, Angina pectoris, kardiale Arrhythmien)
Zerebrovaskuläre Erkrankungen (Aneurysmen, Vaskulitiden, Schlaganfall)

### Psychotherapie und andere nichtmedikamentöse Behandlungsformen

Grundsätzlich steht darüber hinaus mittlerweile eine Vielzahl verhaltenstherapeutisch orientierter, wirksamer nichtmedikamentöser Behandlungsmethoden der ADHS zur Verfügung [19], deren Validierung für das höhere Lebensalter jedoch noch aussteht. Grundsätzlich lässt allerdings bereits die hohe Belastung älterer ADHS-Patienten mit psychiatrischen Komorbiditäten vermuten, dass ihnen in vielen Fällen *lege artis* – unabhängig vom Vorliegen einer ADHS – eine Verhaltenstherapie angeboten werden sollte, um eine Verbesserung der Krankheitsverarbeitung sowie der Funktionsfähigkeit im Alltag und eine Reduktion der Symptomlast zu erreichen. Bei älteren Patienten könnten zudem Behandlungsansätze sinnvoll sein, die die Akzeptanz und gezielte Kompensation altersbedingter Defizite fördern. Eine altersangepasste Psychoedukation ist hier ein essenzieller Behandlungsbaustein. Auch achtsamkeitsbasierte Interventionen können leitliniengerecht in das therapeutische Gesamtkonzept integriert und z. B. mit einer Verhaltenstherapie kombiniert werden. Andere Behandlungsverfahren, z. B. Neurofeedback, sollten angesichts mangelnder Daten derzeit nur in individuellen Einzelfällen und bei fehlendem Ansprechen auf andere Therapien erwogen werden. Darüber hinaus liegen vielversprechende Belege dafür vor, dass sportliche Aktivität signifikant zur Reduktion einer ADHS-Symptomatik beitragen könnte [20, 21]. Ob durch Sportinterventionen auch spezifisch die Kernsymptomatik einer ADHS adressiert werden kann, ist derzeit noch unsicher [7]. Obwohl hier also noch größere systematische Untersuchungen hinsichtlich ADHS-spezifischer Effekte notwendig sind, konnten bislang auch keine ungünstigen Effekte kontrollierter sportlicher Aktivität bei ADHS nachgewiesen werden. Gerade ADHS-Patienten im höheren Lebensalter kann jedoch moderate körperliche Aktivität angeboten werden, um einem alterskorrelierten Abbau der körperlichen Belastbarkeit entgegenzuwirken. Für computergestützte Trainingsprogramme zur Verbesserung bzw. Stärkung der kognitiven Fähigkeiten konnten bei Erwachsenen mit ADHS und betagten gesunden Probanden moderate Effekte nachgewiesen werden, deren klinische Bedeutung allerdings noch unsicher ist [10, 14].

## Ausblick und zukünftiger Forschungsbedarf

Zukünftige Forschung sollte die Sicherheit und Wirksamkeit pharmakologischer Therapien der ADHS im höheren Lebensalter in den Blick nehmen, obgleich ihr Einsatz mit dem Ausscheiden der Betroffenen aus dem Erwerbsleben noch stärker an die jeweilige Lebenssituation und das individuelle Anforderungsprofil anzupassen sein wird. Zudem ist von Interesse, ob sich eine multimodale Therapie der ADHS über die Lebensspanne günstig auf somatische und psychiatrische Komorbiditäten im Alter auswirkt. Hier wäre dann auch zu überprüfen, inwieweit dies einer Frühberentung bzw. Erwerbsminderung entgegenwirken könnte. Darüber hinaus sollte die Wirksamkeit verhaltenstherapeutischer und achtsamkeitsbasierter Interventionen systematisch untersucht werden, um Lebenszufriedenheit und Akzeptanz der Betroffenen zu fördern und eine möglichst selbstständige Lebensführung zu unterstützen. Möglicherweise werden auch digitale Gesundheitsanwendungen bei älteren Patienten in Zukunft eine größere Rolle spielen.

## Fazit für die Praxis

- Auch im höheren Lebensalter ist von einer vergleichsweise hohen Prävalenz der ADHS auszugehen. Die Betroffenen leiden an klinisch relevanten Funktionsstörungen und einer hohen Belastung mit psychiatrischer Komorbidität.
- Es existieren Hinweise, dass eine ADHS mit Persistenz im Erwachsenenalter das Risiko für eine neurodegenerative Erkrankung erhöht.
- Eine ADHS kann auch im höheren Lebensalter zuverlässig diagnostiziert werden. Der differenzialdiagnostischen Abgrenzung gegenüber erworbenen kognitiven Störungen ist hierbei Rechnung zu tragen.
- Eine Behandlung der ADHS mit Stimulanzien im höheren Lebensalter kann zu einer signifikanten Besserung der Symptomatik führen. Dies muss gegenüber möglichen Nebenwirkungen und Interaktionen mit anderen Medikamenten unter Berücksichtigung körperlicher Vorerkrankungen im Rahmen einer Einzelfallentscheidung abgewogen werden.
- Die psychiatrischen Komorbiditäten älterer Patienten mit ADHS stellen oft eine Indikation zur verhaltenstherapeutischen Behandlung dar. Hierbei können altersangepasste ADHS-spezifische Methoden der Akzeptanzförderung und der selektiven Kompensation von Defiziten eine wichtige Ergänzung darstellen.
